# Polysomnographic parameters as early as one week after detoxification could predict risk of relapse among detoxified opiates misuse patients over six months follow up period

**DOI:** 10.1192/j.eurpsy.2021.1508

**Published:** 2021-08-13

**Authors:** A. Rady, J. Mekky, A. Elsheshai

**Affiliations:** 1 Psychiatry, Alexandria University School of Medicine, Alexandria, Egypt; 2 Neurology, Alexandria University school of Medicine, Alexandria, Egypt; 3 Psychiatry, El Mamoura psychiatric hospital, Alexandria, Egypt

**Keywords:** protracted abstinence, polysomnography, opiate, Relapse

## Abstract

**Introduction:**

Protracted abstinence syndrome represent group of attenuated psyc that lead to a persistant sense of discomfort among misuse patients after detoxification and may last for some months.Poor sleep in terms of duration and quality is one of the major symptoms of protracted abstinence syndrome

**Objectives:**

To assess polysomnography parameters as potential risk for relapse over six months

**Methods:**

60 male patients with heroin misuse according to DSM V have been recruited immediately after detoxification phase, they were not receiving other psychactive substances or medications, polysomnography was done in the second week after detoxification to allow washout of medications used during detoxification and then a monthly sleep assessment through sleep diary and daytime sleepiness using visual analogue scale. Relapse was prooved through urine test.

**Results:**

Sample contained 60 male patients with heroin misuse disorder, detoxified successfully with a mean age 35.47±7.32 and addiction severity index total score 3.21±0.22, polysomnography was done to all sample patients one week after detoxification, 20% relapsed by the third month, rising to 30% by the six month. NREM stages I and II, both limb movement and arousal indices showed significant differnce between relapsed and non-relapsed patients.

**Conclusions:**

Sleep disturbance is common among detoxified heroin misuse patients. Polysomnographic parameters such as percentage of NREM I and I, arousal index and limb mouvement index can potentially predict future relpase over six month follow up period.

